# Use of epinephrine combined with dental anesthetics in hypertensive patients during dental treatment

**DOI:** 10.4317/jced.63825

**Published:** 2026-04-25

**Authors:** Alan Garcia Essado, Daniele Morais Dias, Gabriella Tosta Silva, Luciano de Souza Gonçalves, Vinícius Rangel Geraldo Martins, Rodrigo Galo, Marcelo Rodrigues Pinto

**Affiliations:** 1 Department of Biomaterials, University of Uberaba, Uberaba, MG, Brazil; 2 Department of Dental Materials and Prosthesis, School of Dentistry of Ribeirão Preto, University of São Paulo, Ribeirão Preto, SP, Brazil; 3 Department of Conservative Dentistry, Federal University of Rio Grande do Sul, Brazil

## Abstract

**Background:**

The use of local anesthetics containing epinephrine is routine in dental practice, although concerns persist regarding their safety in patients with cardiovascular disease.

**Material and Methods:**

This prospective study was designed to evaluate the effects of 2 percent mepivacaine with 1:100,000 epinephrine on hemodynamic stability in individuals with con-trolled hypertension. Sixty-five individuals aged 20 to 60 years were monitored using automated blood pressure and pulse oximetry devices before anesthesia (t0'), five minutes following injection (t5), and thirty minutes after administration (t30). Systolic and diastolic blood pressure, heart rate, and oxygen saturation were assessed.

**Results:**

No significant alterations were detected at any time point in either hypertensive or normotensive subjects.

**Conclusions:**

Epinephrine at a 1:100,000 concentration combined with 2 percent mepivacaine does not compromise short-term hemodynamic stability in patients with controlled hypertension undergoing routine dental extractions.

## Introduction

Several vasoconstrictors are currently used in dentistry, and epinephrine is the most widely used (1 - 3). Beyond its well-known role in emergencies such as anaphylactic shock, it is routinely incorporated into local anesthetic formulations to improve their clinical performance. By slowing systemic absorption at the injection site, epinephrine reduces the risk of toxicity and promotes deeper and longer-lasting anesthesia for both infiltration and nerve block techniques (4 , 5). Despite these clear advantages, health professionals often hesitate to use it in patients with hypertension or other cardiovascular disorders (6). Traditional recommendations advise caution when using epinephrine on individuals with heart disease, uncontrolled or poorly managed hypertension, uncontrolled hyperthyroidism, or uncontrolled diabetes. Concentrations stronger than 1:100,000 have historically been considered as potentially unsafe for these patients, and a maximum dose of 0.04 mg is generally advised for individuals with cardiovascular disease (7 - 9 , 5). Nevertheless, several authors emphasize that epinephrine can be safely administered to hypertensive patients when dosing limits are respected (10 - 13). The cardiovascular and hemodynamic effects associated with dental anesthesia have been discussed for decades (14 - 17). Current evidence suggests that the physiological stress caused by pain and anxiety and the subsequent release of endogenous catecholamines poses a greater risk to cardiac patients than the low dose of exogenous epinephrine typically used in dentistry (18 , 19). Insufficient vasoconstriction can compromise the quality and duration of anesthesia, which can increase patient discomfort and elevate cardiovascular demand during treatment (16 , 20 , 21). Thus, effective local anesthesia relies not only on the anesthetic agent, but also on appropriate vasoconstriction, as well as on the clinician's technical skill and understanding of anatomical variations (22 - 24). Given these considerations, evaluating the true hemodynamic impact of epinephrine in controlled hypertensive patients is essential for guiding clinical decisions. The present study assessed the cardiovascular response of hypertensive individuals undergoing dental extraction with 2 percent mepivacaine combined with epinephrine at a concentration of 1:100,000 (MepEp100). The null hypothesis is that the anesthetic formulation produces no clinically significant alterations in blood pressure, heart rate, or oxygen saturation at any of the monitored time points.

## Material and Methods

This prospective comparative study involved evaluating changes in blood pressure (BP), heart rate (HR), and oxygen saturation (SpO2) before and after administering a local anesthetic containing a vasoconstrictor (epinephrine) during extractions performed at the Dentistry Clinic of Uberaba. The research participants were between 20 and 60 years old and were recruited according to the inclusion criteria of being hypertensive patients with the disease controlled by medication. The control group consisted of patients in good general health with no history of hypertension. This study was conducted and reported in accordance with the Declaration of Helsinki and was approved by the Ethics Committee of the University of Uberaba (protocol No.3.382.751). All participants underwent local anesthesia with 2% mepivacaine combined with epinephrine 1:100,000 (MepEp100), and signed a free and informed consent form. The volume of administered MepEp100 ranged from 1.8 mL (1 tube) to 5.4 mL (3 tubes). Patients who complied with the inclusion criteria for minor oral surgical procedures but did not consent to participate in the study, as well as patients with uncontrolled blood pressure, were excluded from the study. A reflux carpule syringe was used in the study and control groups to prevent inadvertent intravascular injection. The parameters PA, HR, and SpO2 were checked and recorded 5 minutes before the anesthetic intervention, to assess possible interference with the references due to psychogenic effects, and 5 minutes after the anesthetic intervention to allow for the absorption of 2% mepivacaine hydrochloride. The next measurement occurred 30 minutes after the anesthetic intervention, corresponding to the plasma half-life of 2% mepivacaine hydrochloride. An emergency medical kit was made available to manage emergencies that could arise. The BP, HR, and SpO2 measurements were performed by the calibrated primary investigator, who was assisted by a member of the nursing team. Data were obtained using an electronic digital blood pressure monitor (Hem-612, Omron, São Paulo, Brazil) and a digital pulse oximeter (CMS50D, Contec Medical Systems Co., LTD., China). Data analysis was performed using the GraphPad Prism (SPSS Inc., Chicago, IL, USA). The data were subjected to D'Agostino-Pearson normality tests and analysis of variance (ANOVA). A p-value less than 0.05 was considered significant.

## Results

The evaluated parameters are summarized in Table 1.

Table 1

**Figure 1:**
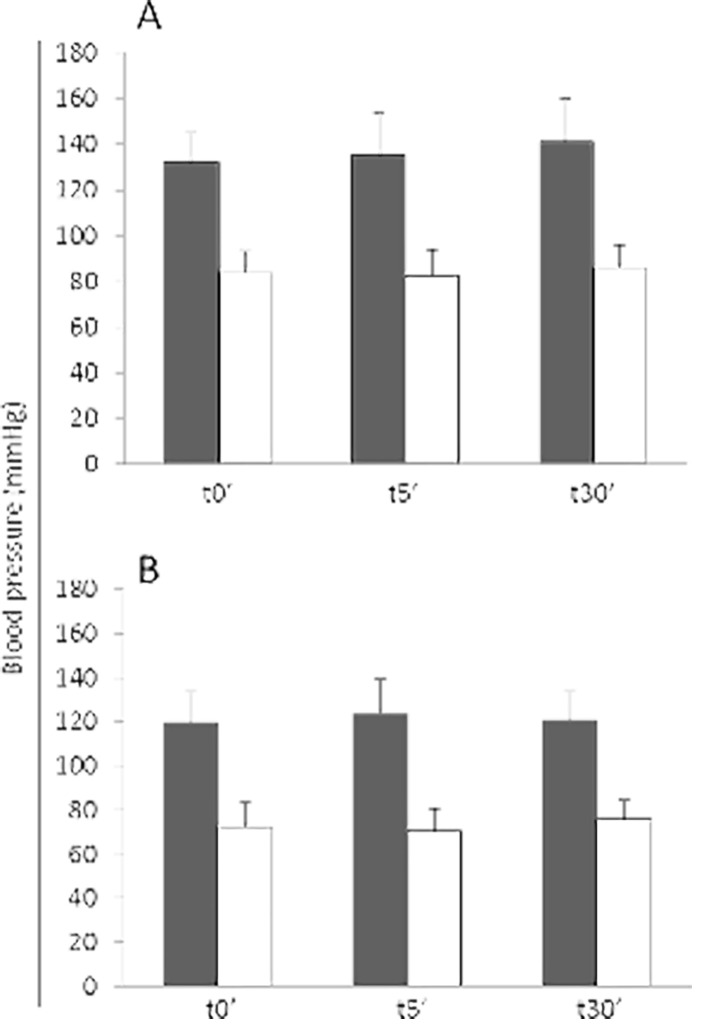
Systolic and diastolic blood pressure during dental treatment with MepEp100 local anesthesia. The graphs represent the blood pressure profiles obtained for hypertensive (A) and non-hypertensive (B) patients. No significant changes were observed in either group (p &lt; 0.05). Time t0’ represents the measurement 5 min before anesthesia administration. Systolic and diastolic blood pressures are represented by the gray and white bars, respectively.

The heart rate data showed that the variations observed over time (t0', t5', and t30') were not significant for the control group (normotensive, p = 0.1134) or the experimental group (hypertensive, p = 0.5862). The mean heart rate for the normotensive individuals before Mep2E100 administration was 70.07 ± 8.27 bpm. After 5 minutes, the frequency increased slightly (74.71 ± 9.60 bpm) and returned to baseline after 30 minutes (70.14 ± 7.99 bpm). For hypertensive patients, the variations were minimal, with values of 73.97 ± 13.07 bpm (t0'), 73.69 ± 11.03 bpm (t5'), and 71.31 ± 11.82 bpm (t30'). Systolic blood pressure (SBP) values for hypertensive patients were 132.4 ± 12.66 mmHg (t0'), 135.5 ± 18.02 mmHg (t5'), and 141.2 ± 18.86 mmHg (t30'); for normotensive individuals, they were 119.4 ± 14.43 mmHg (t0'), 123.8 ± 15.46 mmHg (t5') and 120.7 ± 13.1 mmHg (t30') (Fig. 1).

The observed increase in SBP after MepEp100 injection was not significant for either normotensive (p = 0.5526) or hypertensive (p= 0.1005) subjects. No sudden variations diastolic blood pressure (DBP) were observed before, during, or the administration of MepEp100 for either normotensive patients [72.32 ± 11.41 mmHg (t0'), 70.42 ± 9.77 mmHg (t5'), and 75.84 ± 8.81 mmHg (t30')], or hypertensive patients, whose values were 83.97 ± 9.61 mmHg (t0'), 82.64 ± 11.41 mmHg (t5'), and 85.94 ± 10.0 mmHg (t30') (Fig. 1). Oxygen saturation (SpO2) data showed, over time, normotensive participants had better saturation rates than hypertensive individuals. However, the SpO2 variations observed in each group were not significant. The percentage values obtained for the control group were 98.0 ± 1.02 (t0'), 97.71 ± 1.08 (t5'), and 97.92 ± 0.95 (t30'), while for the experimental group, the values were 96.53 ± 2.3 (t0'), 96.09 ± 2.38 (t5'), and 96.75 ± 2.08 (t30').

## Discussion

The use of local anesthetics combined with vasoconstrictors, particularly epinephrine, has long been a topic of discussion among dental and medical professionals. Although these solutions are well-established in daily practice, concerns about potential cardiovascular effects continue to influence clinical decision-making (6 , 7 , 9). Much of this concern stems from caution surrounding exogenous epinephrine. However, the literature consistently shows that hemodynamic fluctuations observed during dental care are more commonly related to stress, anxiety, and the consequent release of endogenous catecholamines than to the small amounts of vasoconstrictor present in anesthetic formulations (15 , 18 - 20). This study contributes to the existing body of evidence by demonstrating that low-concentration epinephrine is safe for patients with controlled hypertension undergoing dental extraction. In the evaluated population, none of the vital parameters showed a significant change at the different measurement times. Systolic and diastolic blood pressure, heart rate, and oxygen saturation remained stable in both normotensive and hypertensive individuals. These results are consistent with those of previous investigations involving cardiac and hypertensive patients, which also reported no clinically relevant cardiovascular alterations associated with the use of anesthetic solutions containing epinephrine (1 , 2 , 10 , 11 , 13). These findings highlight a critical concept in dental anesthesiology. Cardiovascular safety is determined not by the anesthetic salt used, but by the concentration and total dose of the vasoconstrictor. Epinephrine improves anesthetic performance by reducing systemic absorption and prolonging the anesthetic effect (5 , 15 , 16). Adequate anesthesia also prevents painful stimuli that would otherwise trigger substantial endogenous catecholamine release (18 , 20). In many clinical scenarios, avoiding the vasoconstrictor results in inadequate analgesia, exposing the patient to greater physiological stress than a controlled dose of exogenous epinephrine would. Clinical recommendations suggest that patients with cardiovascular disease should not receive more than 0.04 mg of epinephrine, which corresponds to approximately three anesthetic cartridges containing a 1:100,000 concentration of epinephrine (5 , 7 , 9). The maximum amount administered in this study was 5.4 mL, aligning with this guideline and failing to produce significant hemodynamic changes. These results are consistent with those of trials evaluating similar volumes and concentrations in hypertensive patients, which also reported stable cardiovascular responses (1 , 2 , 12). These findings are relevant to a range of dental procedures that require profound anesthesia. Restorative treatments, periodontal therapy, minor oral surgeries, and emergency interventions all benefit from the predictable analgesic effect of epinephrine. Studies that have monitored hemodynamic parameters during routine dental procedures have consistently demonstrated stable results when epinephrine-containing anesthetic solutions are used appropriately (15 , 21 , 25 , 26). Although alternative vasoconstrictors, such as felipressin, are sometimes considered for patients with specific contraindications, the literature presents inconsistent results regarding their hemodynamic behavior (27 - 30). In contrast, epinephrine at low concentrations is one of the most predictable and well-supported vasoconstrictors in terms of safety and clinical effectiveness. Therefore, decisions about its use should be based on an understanding of the appropriate dose and concentration rather than its classification as an adrenergic agent. Overall, our findings indicate that using 2 percent mepivacaine with epinephrine at a 1:100,000 concentration did not compromise hemodynamic stability in controlled hypertensive patients. Blood pressure, heart rate, and oxygen saturation remained stable throughout the evaluations, reinforcing the idea that the vasoconstrictor does not pose an additional cardiovascular risk when administered within the recommended limits (15 , 18). These results draw attention to an aspect that is sometimes overlooked in clinical practice. The potential impact of epinephrine depends on the concentration and total amount delivered, not just the presence of a vasoconstrictor in the anesthetic. Therefore, noting only that the anesthetic was "combined with adrenaline" does not accurately describe the patient's exposure. When used properly, epinephrine provides more effective anesthesia, reduces the release of endogenous catecholamines associated with pain or anxiety, and contributes to a more stable cardiovascular response during dental care. The data obtained here also support the null hypothesis because no meaningful variations were detected across the monitored time points. Both hypertensive and normotensive patients responded similarly to the anesthetic solution, maintaining stable clinical parameters after the injection. This pattern suggests that the small dose of epinephrine commonly used in dental anesthesia is unlikely to impose cardiovascular strain when safety limits are observed (15 , 18). In many cases, the physiological responses triggered by discomfort, fear, or anticipation of the procedure may pose a greater challenge to the cardiovascular system than the vasoconstrictor itself. This study has limitations that should be considered when interpreting the results. First, group allocation was not randomized, which may introduce selection bias. Due to the clinical nature of the intervention, blinding was not feasible and should be acknowledged as a potential source of measurement bias, even though calibrated devices were used. The sample was restricted to controlled hypertensive patients aged 20 to 60 years, which limits extrapolation to older individuals and those with higher cardiovascular risk. Only one type of dental procedure was evaluated, so the results may not reflect hemodynamic responses during longer, more invasive, or anxiety-provoking treatments. Hemodynamic monitoring was limited to 30 minutes after anesthetic administration; therefore, delayed effects cannot be excluded. Anxiety, pain perception, and surgical difficulty were not quantitatively measured and may have acted as confounding variables. Intermittent rather than continuous monitoring may have failed to detect transient peaks in blood pressure or heart rate. Finally, no a priori sample size or power calculation was performed for hemodynamic outcomes, which increases the risk of type II error.

## Conclusions

Within the limitations of this study, administering 2 percent mepivacaine with epinephrine at a concentration of 1:100,000 did not result in significant changes in blood pressure, heart rate, or oxygen saturation in normotensive or controlled hypertensive patients undergoing dental extraction. These results suggest that when administered within the recommended dosage limits, this anesthetic formulation does not adversely affect short-term hemodynamic stability. However, these findings should be interpreted with caution and cannot be extrapolated to patients with uncontrolled hypertension or higher cardiovascular risk, nor to more invasive or prolonged procedures. Further research with randomized designs, continuous monitoring, and a wider range of patient populations is necessary to enhance our understanding of these effects.

## Figures and Tables

**Table 1:** Parameters assessed during dental treatment.

Parameters	Group	Mean ± SD (t0’)	Mean ± SD (t5’)	Mean ± SD (t30’)	P value
Systolic blood pressure	ControlHypertensive	119.4 ± 14.43132.4 ± 12.66	123.8 ± 15.46135.5 ± 18.02	120.7 ± 13.1141.2 ± 18.86	0.55260.1005
Diastolic blood pressure	ControlHypertensive	72.32 ± 11.4183.97 ± 9.61	70.42 ± 9.7782.64 ± 11.41	75.84 ± 8.8185.94 ± 10.0	0.07360.4058
Heart rate	ControlHypertensive	70.07 ± 8.2773.97 ± 13.07	74.71 ± 9.6073.69 ± 11.03	70.14 ± 7.9971.31 ± 11.82	0.07690.5862
O2 saturation (%)	ControlHypertensive	98 ± 1.0296.53 ± 2.3	97.71 ± 1.0896.09 ± 2.38	97.92 ± 0.9596.75 ± 2.08	0.57710.4803

1
